# Characterization of a new WHIM syndrome mutant reveals mechanistic differences in regulation of the chemokine receptor CXCR4

**DOI:** 10.1016/j.jbc.2021.101551

**Published:** 2021-12-30

**Authors:** Jiansong Luo, Francesco De Pascali, G. Wendell Richmond, Amer M. Khojah, Jeffrey L. Benovic

**Affiliations:** 1 Department of Biochemistry and Molecular Biology, Sidney Kimmel Medical College, Thomas Jefferson University, Philadelphia, Pennsylvania, USA; 2 Section of Allergy and Immunology, Department of Medicine, Rush University Medical Center, Chicago, Illinois, USA; 3 Division of Allergy, Immunology and Rheumatology, Ann & Robert Lurie Children’s Hospital of Chicago, Chicago, Illinois, USA

**Keywords:** cell signaling, chemokine, chemokine receptor, endocytosis, extracellular-signal-regulated kinase (ERK), G-protein-coupled receptor (GPCR), pepducin, protein degradation, protein phosphorylation, AIP4, atrophin-1-interacting protein 4, Bis I, bisindolylmaleimide I, BRET, bioluminescence resonance energy transfer, DMEM, Dulbecco’s modified Eagle’s medium, ELISA, enzyme-linked immunosorbent assay, ERK, extracellular signal-regulated kinase, FBS, fetal bovine serum, fs, frame shift, G-CSF, granulocyte colony stimulating factor, GPCR, G-protein-coupled receptor, GRK, G-protein-coupled receptor kinase, HBSS, Hank’s buffered saline solution, PBS, phosphate-buffered saline, PKC, protein kinase C, pS, phosphoserine, Ser, serine, TBS, Tris-buffered saline, TBST, Tris-buffered saline with Tween, WBC, white blood cell, WT, wild type

## Abstract

WHIM syndrome is a rare immunodeficiency disorder that is characterized by warts, hypogammaglobulinemia, infections, and myelokathexis. While several gain-of-function mutations that lead to C-terminal truncations, frame shifts and point mutations in the chemokine receptor CXCR4 have been identified in WHIM syndrome patients, the functional effect of these mutations are not fully understood. Here, we report on a new WHIM syndrome mutation that results in a frame shift within the codon for Ser339 (S339fs5) and compare the properties of S339fs5 with wild-type CXCR4 and a previously identified WHIM syndrome mutant, R334X. The S339fs5 and R334X mutants exhibited significantly increased signaling compared to wild-type CXCR4 including agonist-promoted calcium flux and extracellular-signal-regulated kinase activation. This increase is at least partially due to a significant decrease in agonist-promoted phosphorylation, β-arrestin binding, and endocytosis of S339fs5 and R334X compared with wild-type CXCR4. Interestingly, there were also significant differences in receptor degradation, with S339fs5 having a very high basal level of degradation compared with that of R334X and wild-type CXCR4. In contrast to wild-type CXCR4, both R334X and S339fs5 were largely insensitive to CXCL12-promoted degradation. Moreover, while basal and agonist-promoted degradation of wild-type CXCR4 was effectively inhibited by the CXCR4 antagonist TE-14016, this had no effect on the degradation of the WHIM mutants. Taken together, these studies identify a new WHIM syndrome mutant, CXCR4-S339fs5, which promotes enhanced signaling, reduced phosphorylation, β-arrestin binding and endocytosis, and a very high basal rate of degradation that is not protected by antagonist treatment.

WHIM syndrome is a rare, genetic disease, which is named for its four key clinical manifestations, Warts, Hypogammaglobulinemia, recurrent Infections, and Myelokathexis (1). WHIM syndrome mainly results from heterozygous gain-of-function mutations in the chemokine receptor CXCR4, a 352 amino acid G-protein-coupled receptor (GPCR) that contains a Ser/Thr-rich C-terminal tail that is a primary region of regulation. CXCR4 is broadly expressed and has an important role in the immune system (1, 2, 3). Previous studies have identified a total of 11 autosomal dominant mutations in CXCR4 across 105 patients that result in WHIM syndrome with the most prevalent being a truncation after Lys333 (R334X) (1). R334X has been the most extensively characterized mutation and has been shown to have increased agonist (CXCL12)-promoted signaling, increased G protein interaction, reduced interaction with GPCR kinase 6 (GRK6) and β-arrestin2, and impaired receptor internalization (1, 4, 5, 6, 7). An S338X mutation has also been extensively studied and has many properties similar to R334X with increased signaling and G protein interaction and decreased β-arrestin2 binding and receptor internalization (8, 9, 10, 11, 12). Another WHIM mutation that has been well studied is E343K, which has increased signaling and reduced receptor phosphorylation and internalization (13, 14, 15). Interestingly, one WHIM syndrome patient has also been identified with a defect in the protein kinase GRK3 resulting in increased CXCL12-promoted signaling and impaired desensitization and internalization (16, 17).

CXCR4 has been extensively investigated and implicated in several diseases besides WHIM syndrome including acquired immunodeficiency syndrome (AIDS), where it serves as a coreceptor for human immunodeficiency virus (HIV), and cancer, where it has a role in metastasis (18, 19). Moreover, CXCR4 mutations found in WHIM syndrome also occur in ∼30% of the cases of the plasma cell cancer Waldenstrom’s macroglobulinemia (20, 21). Recent cancer genome deep sequencing efforts have also revealed a higher frequency of mutations in CXCR4 in some tumor types, such as diffuse large B-cell lymphoma, uterine corpus endometrial carcinoma, and skin cutaneous melanoma (22). The C-terminal mutations in CXCR4 that occur in WHIM syndrome appear to disrupt the normal mechanisms involved in regulating CXCR4 function, which include agonist-dependent phosphorylation of the receptor by GRKs and protein kinase C (PKC), desensitization mediated by β-arrestin binding, ubiquitination by the E3 ubiquitin ligase AIP4, endocytosis, and sorting to lysosomes where the receptor is degraded (14, 23, 24, 25, 26). CXCR4 has also been shown to regulate several signaling pathways primarily *via* its interaction with G_i_ although it can also activate G_13_ (18, 27). While C-terminal truncations and mutations in CXCR4 have been shown to enhance CXCR4 signaling and lead to WHIM syndrome, we still have an incomplete picture of how these changes alter CXCR4 function.

Here we focused on understanding the characteristics of a new WHIM syndrome mutation in CXCR4 that we identified that contains a frame shift in the codon for Ser339 resulting in five additional residues (S339fs5). Patients that have this mutation exhibit many characteristics of WHIM syndrome including recurrent warts, human papillomavirus-associated cervical dysplasia, frequent respiratory tract infections, and leukopenia with bone marrow biopsy evidence of myelokathexis. To better understand the characteristics of S339fs5 that might contribute to its role in WHIM syndrome, we generated a stable cell line expressing S339fs5 and compared its properties with wild-type (WT) CXCR4 and with another WHIM syndrome mutant, R334X. S339fs5 and R334X have a defect in the normal mechanisms involved in CXCR4 regulation including decreased agonist-promoted phosphorylation, β-arrestin binding, and endocytosis ultimately leading to enhanced signaling compared to WT CXCR4. S339fs5 also appears to have a higher basal rate of degradation compared with R334X and WT CXCR4 suggesting that S339fs5 levels might be lower compared with WT CXCR4 in these patients.

## Results

### Phenotypic characterization of a family with WHIM syndrome

**Figure 1:**
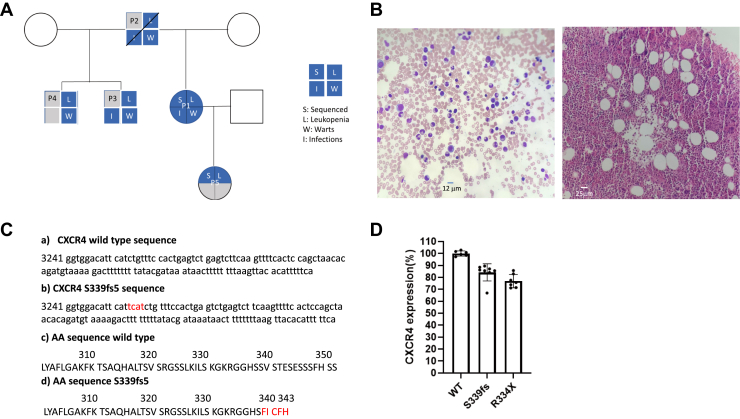
**Characterization of a new WHIM syndrome cohort.***A*, pedigree of a family that has symptoms of WHIM syndrome. *B*, *left panel*—peripheral smear of patient P1; *right panel*—bone marrow biopsy from patient P1 showing myelokathexis. *C*, sequence analysis of *CXCR4* from patient P1 with the altered base pair and amino acid sequences compared with wild-type *CXCR4* shown in *red*. *D*, cell surface expression of WT CXCR4, S339fs5 (S339fs), and R334X as measured by ELISA from 6 to 8 experiments with mean ± SD shown.

Here we report on a new patient cohort containing a mutation in *CXCR4* that results in a frame shift within the codon for Ser339 (S339fs5) (Fig. 1). Data from the index case (P1) show leukopenia with marked neutropenia and decreased class-switched memory B cells with normal quantitative immunoglobulins. Infections, such as other CXCR4 WHIM syndrome variants, include human papillomavirus-associated disease including warts and mucosal intraepithelial lesions. Nonepithelial infections in P1 and her father (P2) are restricted to the respiratory tract. A comparison of the sequence of S339fs5 with other reported WHIM syndrome mutations is shown in Fig. S1.

### Generation of stably overexpressing HEK293 cell lines

To characterize the properties of the new S339fs5 mutation, we generated HEK293 cells stably overexpressing Flag-tagged S339fs5 as well as a stable line overexpressing another WHIM mutant R334X, the most prevalent mutation found in WHIM syndrome patients. ELISA was then used to compare the cell surface expression of S339fs5 and R334X with a line that we had previously developed expressing WT CXCR4 (25). This showed that S339fs5 and R334X were expressed at 75–85% of the level observed in WT CXCR4 expressing cells (Fig. 1*D*).

### Effects of WHIM mutations on CXCR4 signaling

**Figure 2:**
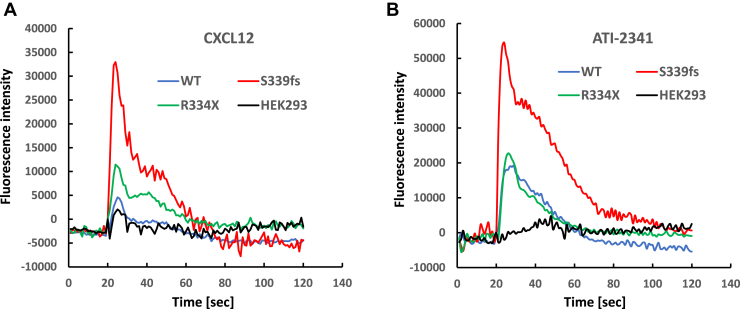
**Analysis of calcium flux in HEK293 cells with or without stable expression of WT CXCR4 or WHIM mutants S339fs5 and R334X.** HEK293 cells grown in a 96 well plate were loaded with Fluo4-AM at 37 °C for 1 h and washed with Hank’s buffer. Cells were stimulated with 50 nM CXCL12 (*A*) or 3 μM ATI-2341 (*B*), a G_i_-biased pepducin for CXCR4, and the fluorescence intensity was monitored over 120 s. These results are representative of four experiments.

Previous studies have noted that CXCR4 mutations involved in WHIM syndrome have enhanced signaling. To compare the signaling of S339fs5 with R334X and WT CXCR4, we initially looked at the ability of the CXCR4 agonist CXCL12 to stimulate a calcium flux. CXCL12 was found to promote a low level of calcium flux in untransfected HEK293 cells due to a low level of endogenous CXCR4 (Fig. 2*A*). This response was modestly increased in cells overexpressing WT CXCR4, a finding that we previously observed in these cells (25). In contrast, calcium flux was increased several fold in cells expressing R334X and almost tenfold in cells expressing S339fs5. We also evaluated the ability of the G_i_-biased CXCR4 pepducin ATI-2341 (28) to stimulate a calcium flux in the various lines. Untransfected HEK293 cells had a low level of calcium flux, cells expressing WT CXCR4 had a much higher level of response to ATI-2341, R334X cells were comparable to WT, and S339fs5 cells were increased approximately threefold compared with WT CXCR4 (Fig. 2*B*). Overall, these studies show that both S339fs5 and R334X have enhanced calcium signaling compared with WT CXCR4 with S339fs5 being the most responsive.

**Figure 3:**
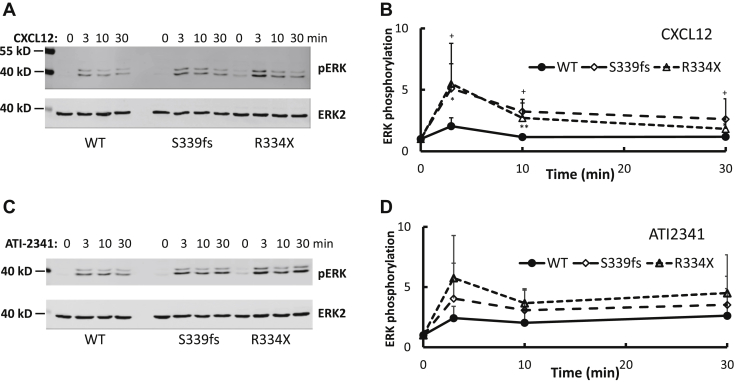
**Analysis of ERK1/2 activation in HEK293 cells stably expressing WT CXCR4 or WHIM mutants S339fs5 and R334X.** HEK293 cells were incubated in serum-free medium for 4 h and then stimulated with 50 nM CXCL12 (*A* and *B*) or 3 μM ATI-2341 (*C* and *D*). Panels *A* and *C* show a representative time course of ERK1/2 phosphorylation while panels *B* and *D* summarize results from six individual experiments as fold over basal with mean ± SD shown. *p* < 0.05 at 3 min, *p* < 0.01 at 10 min and *p* = 0.05 at 30 min when comparing S339fs5 with WT stimulated with CXCL12. *p* < 0.05 at 3, 10, and 30 min when comparing R334X with WT stimulated with CXCL12.

CXCR4 also promotes the phosphorylation of extracellular-signal-regulated kinase (ERK)1/2 through G_i_ and GRK/β-arrestin-dependent pathways (25, 28). Our results show low ERK1/2 activation induced by CXCL12 in untransfected HEK293 cells with a significant increase in cells expressing WT CXCR4 as previously demonstrated (25 and data not shown). Cell lines expressing R334X or S339fs5 had enhanced CXCL12-promoted ERK1/2 activation compared with WT CXCR4 cells with R334X being the most responsive (Fig. 3, *A* and *B*). Similarly, ERK1/2 activation induced by ATI-2341 showed some activation in untransfected HEK293 cells with an increase in cells expressing WT CXCR4 (data not shown). The cell lines expressing R334X or S339fs5 both had enhanced ATI-2341-promoted ERK1/2 activation compared with WT CXCR4 with R334X again being the most active (Fig. 3, *C* and *D*). Taken together, these studies show that both S339fs5 and R334X have enhanced ERK1/2 activation compared with WT CXCR4 with R334X being the most responsive.

### Effects of WHIM mutations on CXCR4 phosphorylation

**Figure 4:**
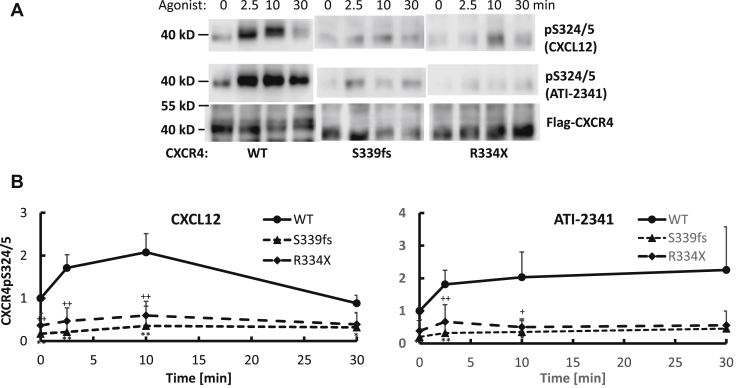
**Analysis of CXCR4 phosphorylation at serines 324 and 325 (pS324/5) in HEK293 cells stably expressing WT CXCR4 or WHIM mutants S339fs5 and R334X.** HEK293 cells were starved in serum-free medium for 4 h and then stimulated with 50 nM CXCL12 or 3 μM ATI-2341. *A*, representative Western blot of CXCR4 detected using a pS324/5 specific antibody or Flag antibody. *B*, data summarizing the quantification of CXCR4-pS324/5 from four individual experiments in cells expressing WT CXCR4, CXCR4-S339fs5, or CXCR4-R334X with mean ± SD shown. *p* < 0.001 at basal, 3 min and 10 min, and *p* < 0.05 at 30 min when comparing S339fs5 with WT stimulated with CXCL12. *p* < 0.005 at basal and 10 min, *p* < 0.001 at 3 min, and *p* < 0.05 at 30 min when comparing R334X with WT stimulated with CXCL12. *p* < 0.001 at basal, *p* < 0.005 at 3 min, and *p* < 0.05 at 10 and 30 min when comparing S339fs5 with WT stimulated with ATI-2341. *p* < 0.01 at basal, 3 and 10 min, and *p* < 0.05 at 30 min when comparing R334X with WT stimulated with ATI-2341.

Increased signaling suggests increased activation of G_i_-dependent pathways possibly due to a decrease in the mechanisms that desensitize or turn-off CXCR4 activation of G_i_. Previous studies support a role for GRK and PKC-mediated phosphorylation of CXCR4 in the desensitization process (25). In particular, CXCL12-mediated phosphorylation of far C-terminal residues such as Ser346-8 by GRK2/3 is thought to promote β-arrestin binding, which functions in the desensitization process (14, 25, 26). While these residues are not present in R334X or S339fs5, Ser324 and Ser325 (S324/5) are present and also contribute to the desensitization process as well as to CXCR4 endocytosis and degradation (23). Thus, we next evaluated CXCL12 and ATI-2341-mediated phosphorylation of S324/5 using phospho-specific antibodies that we previously generated and validated (25). While CXCL12 and ATI-2341 induced rapid and robust phosphorylation of S324/5 in WT CXCR4, there was a lower basal level of phosphorylation and minimal agonist-induced phosphorylation of S339fs5 and R334X (Fig. 4, *A* and *B*). It is also worth noting that we see reduced mobility of WT CXCR4 following agonist treatment due to phosphorylation of the receptor as previously shown (25), while no mobility shift was observed with the WHIM mutants (Fig. 4*A*). Thus, S339fs5 and R334X undergo minimal agonist-promoted phosphorylation of critical residues involved in regulating CXCR4 function.

### Effects of WHIM mutations on β-arrestin binding

**Figure 5:**
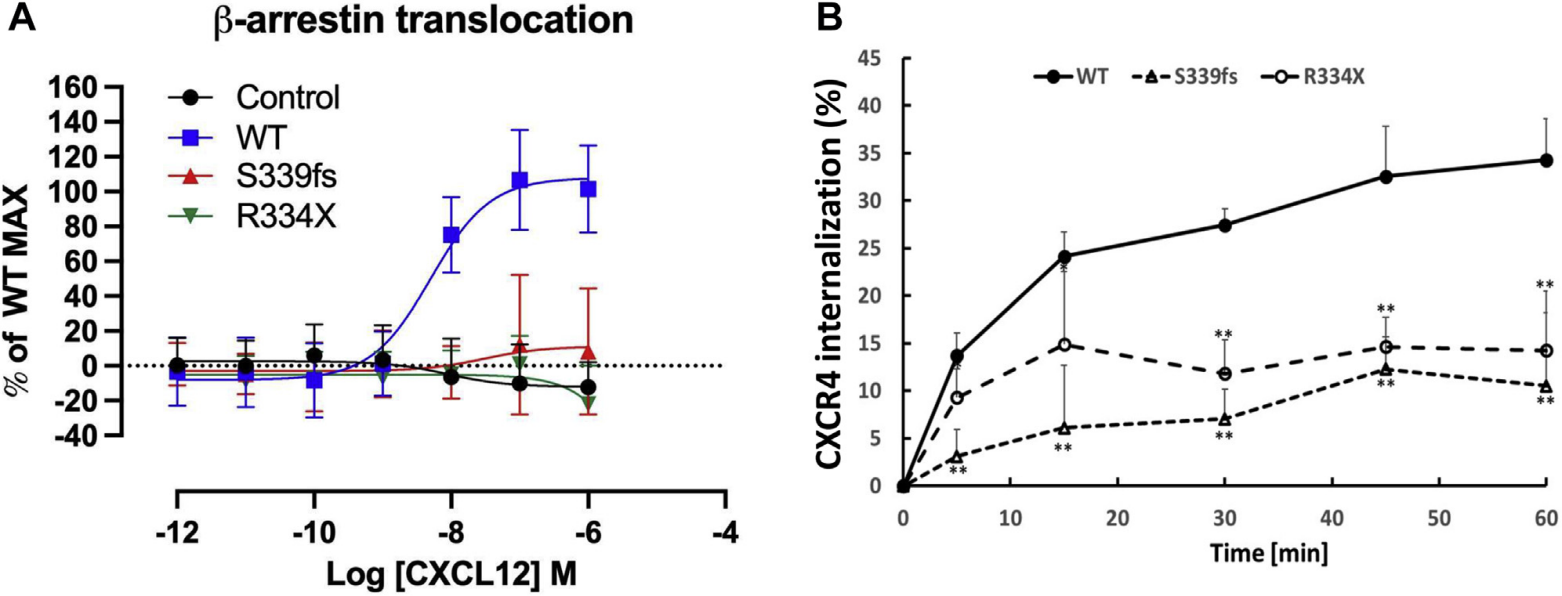
**Analysis of β-arrestin2 translocation and CXCR4 internalization in HEK293 cells expressing WT CXCR4 or WHIM mutants S339fs5 and R334X.***A*, HEK293 cells were transiently transfected with Rluc8-β-arrestin2-Sp1 and M-L-citrine-SH3 with or without WT CXCR4, S339fs5, or R334X and analyzed for CXCL12-dependent BRET 48 h after transfection. The data are from five experiments with mean ± SD shown. *B*, HEK293 cells stably expressing WT CXCR4 or WHIM mutants S339fs5 and R334X grown in a 24-well plate were treated with 50 nM CXCL12 at 37 °C for 0 to 60 min and the cells were fixed, incubated with anti-flag polyclonal antibody, and cell surface receptors detected by chemiluminescence as described in Experimental procedures. The data from at least six individual experiments with mean ± SD shown (∗*p* < 0.05; ∗∗*p* < 0.001).

Previous studies have shown that β-arrestins bind to CXCR4 in an agonist- and phosphorylation-dependent manner and play an important role in regulating CXCR4-mediated signaling (16, 18, 25, 26). To evaluate whether the WHIM mutants have a defect in β-arrestin binding, we utilized a bioluminescence resonance energy transfer (BRET) assay to study the translocation of β-arrestin2 from the cytoplasm to the plasma membrane upon receptor activation (29). Initial studies to validate the assay demonstrated CXCL12-dependent translocation of β-arrestin2 in HEK293 cells expressing WT CXCR4 with no translocation in untransfected cells (Fig. 5*A*). We also did not detect significant translocation of β-arrestin2 in either the S339fs5- or R334X-expressing cells (Fig. 5*A*). Thus, the S339fs5 and R334X WHIM mutants have a defect in β-arrestin binding most likely due to the truncated C-terminus and reduced C-terminal phosphorylation compared with WT CXCR4.

### Effects of WHIM mutations on CXCR4 endocytosis

One important aspect of CXCR4 regulation is agonist-promoted endocytosis, which ultimately is critical in the subsequent sorting and degradation of the receptor. While the mechanism of this endocytosis has not been fully dissected, it has been shown that PKC activation can induce CXCR4 endocytosis *via* a pathway that is likely β-arrestin independent (30). WT CXCR4 was found to undergo rapid CXCL12-induced endocytosis achieving ∼35% after 1 h of agonist treatment (Fig. 5*B*). CXCL12-induced endocytosis of R334X was significantly reduced with a maximum of ∼15% at 1 h while S339fs5 was reduced even further with a maximum of ∼10% (Fig. 5*B*). Since the phosphorylation of S324/5 plays an important role in the endocytic process (23), it is not surprising that agonist-dependent endocytosis of S339fs5 and R334X is significantly ablated given the low level of pS324/5 that was observed (Fig. 4).

### Effects of WHIM mutations on basal and agonist-promoted degradation of CXCR4

**Figure 6:**
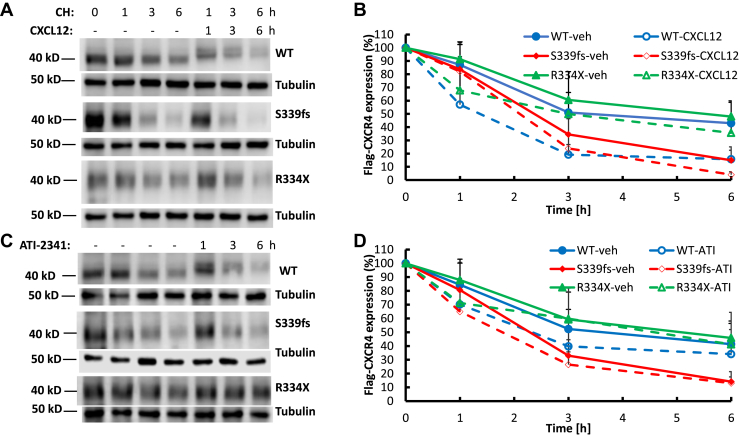
**Analysis of CXCR4 degradation in HEK293 cells stably expressing WT CXCR4 or WHIM mutants S339fs5 and R334X.** Cells grown in a 12 well plate were pre-incubated with 20 μg/ml of cycloheximide (CH) for 10 min and then incubated with or without 50 nM CXCL12 (*A* and *B*) or 3 μM ATI-2341 (*C* and *D*) for 0 to 6 h. Receptor degradation was analyzed by Western blot using an anti-flag polyclonal antibody. *A* and *C*, representative Western blot of CXCR4 after treatment with CXCL12 or ATI-2341 for 0 to 6 h. *B* and *D*, quantification of CXCR4 degradation from eight individual experiments was analyzed using a LI-COR scanner with mean ± SD shown: *p* < 0.01 at 1 and 6 h, and *p* < 0.05 at 3 h when comparing basal WT degradation with CXCL12 stimulated degradation; *p* < 0.01 at 6 h when comparing S339fs5 with WT basal degradation. *p* < 0.01 at 1 and 3 h, and *p* < 0.05 at 6 h when comparing basal WT degradation with ATI-2341 stimulated degradation.

CXCR4 is also regulated by sorting to lysosomes where it is degraded (23, 24). To evaluate potential differences in the degradation of WT, S339fs5, and R334X CXCR4, we first treated the overexpressing cells with cycloheximide for 10 min to inhibit new protein synthesis. We then treated the cells with buffer, CXCL12 or ATI-2341 for 0 to 6 h and assessed CXCR4 levels by immunoblotting. WT CXCR4 undergoes significant basal degradation in this system with a half-life of ∼4-h while CXCL12 significantly increased the rate of degradation with a half-life of ∼1.4 h (Fig. 6, *A* and *B*). ATI-2341 also enhanced the rate of CXCR4 degradation although it was not as significant as observed with CXCL12 (Fig. 6, *C* and *D*). While R334X had a basal rate of degradation similar to WT CXCR4, there was no significant effect of CXCL12 or ATI-2341 treatment (Fig. 6). Interestingly, S339fs5 had a higher basal rate of degradation with a half-life of ∼2.5 h but, similar to R334X, it was largely insensitive to agonist treatment (Fig. 6). Thus, the rates of degradation of WT, R334X, and S339fs5 are significantly different with WT being sensitive to agonist treatment, R334X and S339fs5 being agonist-insensitive, and S339fs5 having a higher basal rate of degradation.

**Figure 7:**
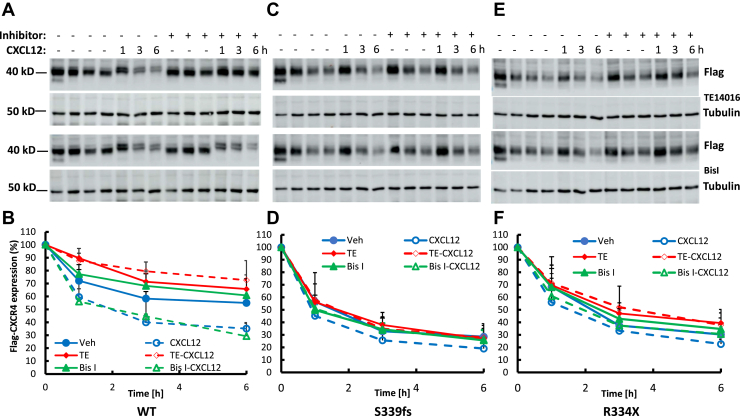
**Effects of a CXCR4 antagonist and PKC inhibitor on CXCR4 degradation in HEK293 cells stably expressing WT CXCR4 or WHIM mutants S339fs5 and R334X.** A time course of CXCL12-promoted CXCR4 degradation in the presence or absence of 1 μM of the CXCR4 antagonist TE14016 or 10 μM of the PKC inhibitor Bis I was performed. Panels *A*, *C*, and *E* show representative Western blots of basal and CXCL12-induced CXCR4 degradation in the presence or absence of TE14016 or Bis I. Panels *B*, *D*, and *F* show quantification of CXCR4 degradation from at least six individual experiments with mean ± SD shown. WT: *p* < 0.01 at 1, 3, and 6 h when comparing basal degradation with CXCL12 stimulated degradation; *p* < 0.01 at 1 h when comparing TE14016 on basal degradation; *p* < 0.01 at 1, 3, and 6 h when comparing TE14016 on CXCL12 stimulated degradation; Bis I has no effect on basal degradation. S339fs5: *p* < 0.01 at 3 and 6 h when comparing basal degradation with CXCL12 stimulated degradation. R334X: *p* < 0.05 at 3 and 6 h when comparing TE14016 on basal degradation; *p* < 0.05 at three and *p* < 0.01 at 6 h when comparing TE14016 on CXCL12 stimulated degradation; Bis I has no effect on basal or CXCL12 stimulated degradation.

To try to better understand potential mechanistic differences between the degradation of WT CXCR4 and the WHIM mutants, we treated cells with the high affinity CXCR4 antagonist TE14016 or the PKC inhibitor Bis I. TE14016 significantly inhibited basal degradation as well as CXCL12-promoted degradation of WT CXCR4 while Bis I partially inhibited basal degradation (Fig. 7, *A* and *B*). In contrast, we did not see a significant effect of TE14016 or Bis I treatment on the degradation of S339fs5 (Fig. 7, *C* and *D*) or R334X (Fig. 7, *E* and *F*). Thus, the higher basal rate of degradation of S339fs5 does not appear to be due to PKC-mediated phosphorylation of the receptor.

## Discussion

In the present study, we report on a new patient cohort containing a mutation in *CXCR4* that results in a frame shift within the codon for Ser339 resulting in the addition of five new residues after Ser338 (CXCR4-S339fs5). To better understand the properties of S339fs5 compared with WT CXCR4 and other WHIM syndrome mutants such as R334X, we established HEK293 cell lines stably expressing WT or mutant CXCR4. S339fs5 had enhanced agonist-promoted signaling, decreased phosphorylation, decreased β-arrestin binding, decreased endocytosis, and an increased rate of basal degradation compared with WT CXCR4. Previous studies reported on a group of six patients that have a different frame shift in CXCR4 within the codon for Ser339 (S339fs3), although this mutation was never characterized. S339fs3 would likely have characteristics similar to the S339fs5 mutation that we identified in the current work, although it is possible that the residues after Ser338 might lead to additional changes in CXCR4 function. Nevertheless, we would anticipate that all patients that have a frame shift within the Ser339 codon would have significantly enhanced signaling, reduced receptor phosphorylation, β-arrestin binding, and endocytosis, and an increased rate of basal degradation that is not altered by CXCR4 antagonists. We speculate that treating these patients with a CXCR4 antagonist could reduce the high level of signaling and help to normalize CXCR4 function.

Previous studies have extensively characterized the mechanisms involved in the regulation of CXCR4. CXCR4 is rapidly phosphorylated following activation by CXCL12 with primary sites being Ser324/5, Ser330, Ser339, and Ser346/7 (14, 24, 25). The primary kinases that mediate phosphorylation of these sites include GRK6 and PKC for Ser324/5, GRK6 for Ser330 and Ser339, and GRK2 and/or GRK3 for Ser346/7 (14, 25, 26). Functional effects of CXCR4 phosphorylation include enhanced β-arrestin binding and desensitization (25, 26), ubiquitination of C-terminal arginines by the E3 ligase AIP4 (24), and endocytosis and sorting of the receptor for degradation (23, 24). Some of these processes have been linked with specific sites of phosphorylation although mechanistic details of this are incomplete. For example, mutation of Ser324/5 to alanine results in a complete loss of agonist-promoted degradation and significant decrease in endocytosis of CXCR4 while S330A mutation results in an ∼50% reduction in degradation and no effect on endocytosis (23). Interestingly, recent studies have also shown that the phosphorylation of far C-terminal residues such as Ser346/7 appears to be critical in the phosphorylation of additional residues including Ser324/5, Ser338/9, and possibly Ser330 (14, 31). This may be due to reduced binding of COMMD3/8 leading to a loss of GRK6 recruitment and reduced phosphorylation of residues that contribute to endocytosis, ubiquitination, and lysosomal degradation (31). While we have not evaluated COMMD3/8 binding, we do see a significant reduction in agonist-promoted phosphorylation of Ser324/5 in S339fs5 and R334X suggesting a reduced ability of GRK6 to phosphorylate these sites (Fig. 4).

Previous studies have also extensively characterized the properties of a few other WHIM syndrome mutants. R334X has increased CXCL12-promoted signaling and increased interaction with G protein (4, 6, 7) as well as a defect in GRK6 and β-arrestin2 binding causing delayed receptor internalization and desensitization (5), although these studies did not evaluate effects on receptor phosphorylation. Our work shows reduced agonist-promoted phosphorylation and degradation of R334X. CXCR4-S338X has increased signaling and G protein interaction and decreased β-arrestin2 binding and receptor internalization (8, 9, 10, 11, 12) so it appears similar to R334X. Interestingly, a gain-of-function E343K mutation in CXCR4 was initially reported in a patient cohort by Liu *et al.* (13). E343K was shown to be defective in agonist-promoted phosphorylation of multiple C-terminal sites including S324/5, S338/9, and S346/7 (14). This mutation was further characterized by Wang *et al.* (15), who found that having an acidic residue at position 343 was essential in maintaining normal function and that mutation to Arg, Lys, or Ala enhanced agonist-promoted signaling and impaired desensitization of CXCR4. While the mechanism of reduced phosphorylation was not determined, the mutation likely results in reduced phosphorylation of C-terminal serines that are normally phosphorylated by GRK2 and/or GRK3 (14, 25, 26) since these kinases preferentially phosphorylate Ser/Thr in an acidotropic environment (32).

Taken together, our studies provide insight on a new mutation in CXCR4 that results in WHIM syndrome and help to define the mechanisms involved in the altered regulation of WHIM mutants including S339fs5 and R334X.

## Experimental procedures

### Materials

HEK293 cells were obtained from Microbix Biosystems, Inc, while Lipofectamine 2000 and Opti-MEM were from Invitrogen. Anti-CXCR4 monoclonal antibodies were obtained from BD Biosciences Pharmingen. Anti-CXCR4 phospho-serine 324/5 (pS324/5) antibodies were previously described (25). Anti-flag-tagged monoclonal and polyclonal antibodies and anti-α-tubulin monoclonal antibodies were obtained from Sigma-Aldrich. The CXCR4 pepducin ATI-2341 was synthesized by Peptide 2.0 Inc.

### Patient information

The index patient (P1) is a 41-year-old female who was found to have leukopenia at birth. Since there was no obvious illness associated with the finding, no further investigation was undertaken at that time. Sinus and ear infections began in grade school and warts were first noted at 12 years of age. At 17 years of age, she was found to have a low white blood cell (WBC) count of 1.4 × 10^3^ and was referred to the local children’s hospital for evaluation. No etiology for the leukopenia was identified. At 17, she had sinus surgery due to recurrent sinus infections. At 26 years of age, she was found to be hypothyroid. At 28 years of age, a bone marrow biopsy was obtained. Myelokathexis was appreciated but no additional diagnosis was made. She was found to have HPV with cervical intraepithelial neoplasia 1 requiring a loop electrosurgical excision procedure. The patient continues to experience recurrent episodes of sinusitis and 2–3 episodes of bronchitis yearly. P1’s father (P2) was found to have neutropenia as a child. Since the family lived on a farm, the low WBC count was attributed to chemical exposure. He had a history of multiple pneumonias, warts, and hypothyroidism. He received multiple doses of granulocyte colony stimulating factor (G-CSF), which were effective at increasing WBC counts. His death at 56 years of age was attributed to a pulmonary embolism. P1 has two half siblings (P3, P4). P3 is a male with a history of neutropenia and extensive hand warts. P4 is a male with neutropenia, warts, and MRSA positive skin infections. Therapy with G-CSF has been effective at increasing WBC counts in P4. P1’s paternal grandfather had polycythemia requiring frequent phlebotomies. P1 has a 15-month-old daughter (P5) who is neutropenic but asymptomatic. *CXCR4* sequencing of P1 and P5 has identified the S339fs5 variant. This work was evaluated by the Ann & Robert Lurie Children’s Hospital of Chicago IRB and found to be exempt (IRB #2021-4860).

### Cell culture and generation of stable cell lines

Flag-tagged human CXCR4 was previously described (24) and was used to generate plasmids for the WHIM mutations S339fs5 and R334X using the following primers: S339fs5: Forward: 5′-TTTCCACTAATCTAGAGGGCCCTATTC-3’; Reverse: 5′- CAGATGAATGAA TGTCCACCTCGCTT-3’. R334X: Forward: 5′-CAAAGGAAAGTGAGAGGTGGACATTC-3’; Reverse: 5′-GAGAGGATCTTGAGGCTG-3’. HEK293 cells were maintained in Dulbecco’s modified Eagle’s medium (DMEM) supplemented with 10% fetal bovine serum (FBS), 25 mM HEPES, pH 7.2, and 0.1 mM nonessential amino acids (complete media) in a 5% CO_2_ humidified incubator at 37 °C. HEK293 cells stably expressing WT CXCR4 were previously described (25) while cell lines expressing S339fs5 and R334X were transfected with the appropriate plasmid and selected and maintained in complete media supplemented with 0.8 mg/ml G418 and penicillin/streptomycin.

### Detection of CXCR4 phosphorylation

Cells were plated into 12-well plates and cultured for 24 h. Cells were starved with serum-free DMEM for 4 h prior to stimulation with agonist. Cells were then washed twice with ice-cold phosphate buffered saline (PBS) and lysed with buffer containing 20 mM HEPES, pH 7.2, 10 mM EDTA, 150 mM NaCl, 1% Triton X-100, and one tablet each of minicomplete protease inhibitor and PhosSTOP phosphatase inhibitor (Roche) per 10 ml at 4 °C on a rocker for 30 min. The lysates were cleared by centrifugation at 14,000 rpm in an Eppendorf centrifuge for 20 min at 4 °C. Total protein in cell lysates was measured using the Bio-Rad Protein Assay. An equal amount of total protein was electrophoresed on a 10% SDS polyacrylamide gel, transferred to nitrocellulose, and immunoblotted with the following primary antibodies: polyclonal anti-pS324/5-CXCR4 (1:1000) and either monoclonal anti-CXCR4 (1:1000) or polyclonal anti-flag (1:2000). Blots were washed extensively with Tris-buffered saline (TBS) plus 0.02% Tween 20 (TBST), incubated with a horseradish peroxidase–labeled secondary antibody, washed with TBST, and then proteins were detected using chemiluminescence. The blots were stripped and reprobed using an antitubulin monoclonal antibody. Western blots were visualized and quantified using a C-DiGit Blot Scanner and Image Studio Software (LI-COR).

### ERK activation assays

HEK293 cells expressing Flag-tagged WT or mutant CXCR4 at ∼90% confluence in 12-well plates were serum starved for 4 h and then stimulated with either 50 nM CXCL12 or 3 μM ATI-2341 and washed once with ice cold PBS. Lysis buffer (1% Triton X-100, 20 mM HEPES, pH 7.2, 150 mM NaCl, 10 mM EDTA, phosphatase inhibitor, and protease inhibitor) was added on ice, the cells were rocked at 4 °C for 30 min, and debris was cleared by centrifugation at 14,000 rpm for 20 min. Equal amounts of whole cell lysate were separated by electrophoresis on a 10% SDS polyacrylamide gel, transferred to nitrocellulose, and proteins were detected by immunoblotting. Nitrocellulose membranes were blocked for 1 h at room temperature in a 1:3 dilution of ODYSSEY blocking buffer (LI-Cor Biosciences) and a mixture of primary antibodies directed at ERK2 (monoclonal, Santa Cruz) and phospho-ERK1/2 (polyclonal, Cell Signaling Technologies) in 100% ODYSSEY blocking buffer were incubated with the nitrocellulose overnight at 4 °C. Nitrocellulose membranes were washed three times for 15 min with TBST. The membranes were then incubated for 1 h at room temperature with a mixture of goat anti-rabbit Alexa Fluorophore 680 conjugated (LI-Cor Biosciences) and goat anti-mouse IRDye 800 conjugated (LI-Cor Biosciences) antibodies. Following a 1 h incubation, the membranes were washed with TBST three times for 20 min each. Fluorescence was detected simultaneously using the ODYSSEY infrared imaging system (LI-Cor Biosciences). Fluorescence intensity of phosphorylated ERK1/2 was normalized to total ERK2 fluorescence, and data are represented as fold-increase over basal ± SEM.

### Measurement of intracellular calcium mobilization

HEK293 cells stably expressing CXCR4 were plated at 20,000 to 25,000 cells/well in 100 μl of media into poly-l-lysine (Sigma) coated 96-well plates and incubated at 37 °C overnight. Medium was aspirated and cells were loaded with 2.5 μM Fluo-4 AM in 50 μl Hanks buffered saline solution (HBSS) containing 10 mM HEPES, pH 7.2, and 0.25% pluronic (Life Tech). Plates were incubated at room temperature for 1 h in the dark, the dye was aspirated, and the plate was washed with HBSS one time. Cells were stimulated with 50 nM CXCL12 or 3 μM ATI-2341 in 100 μl HBSS, and the calcium flux was monitored with Clariostar Plus (BMG Labtech), a multimode microplate time-resolved fluorescence reader.

### Analysis of β-arrestin translocation using BRET

We used a β-arrestin translocation assay to monitor the movement of a BRET donor-tagged β-arrestin2 from the cytoplasm to the plasma membrane upon GPCR activation as previously described (29). Briefly, 100,000 HEK293 cells/well in a 96-well opaque microplate (PerkinElmer) were transiently cotransfected with the BRET biosensors Rluc8-β-arrestin2-SP1 (25 ng/well) and M-L-Citrine-SH3 (500 ng/well) with or without 20 ng/well of Flag-CXCR4, Flag-CXCR4-S339fs5, or Flag-CXCR4-R334X using Metafectene Pro (Biontex). Forty-eight hours after transfection, cells were stimulated with increasing concentrations of CXCL12 (ProSpec) in the presence of 5 μM Renilla luciferase coelenterazine H (Cayman Chemical Company) for 20 min. Signals were recorded with a Tecan Infinite F500 plate reader.

### Analysis of cell surface expression and internalization of CXCR4

Cell surface expression of Flag-tagged WT and mutant CXCR4 was measured by ELISA as previously described (33). Briefly, HEK293 cells stably expressing Flag-tagged CXCR4 were split into 24-well plates coated with poly-l-lysine. The following day, the medium was aspirated and cells were fixed for 10 min at room temperature with 3.7% formaldehyde in TBS. Cells were washed three times with TBS and then blocked for 45 min with 1% bovine serum albumin in TBS. Cells were then incubated for 1 h with a rabbit polyclonal Flag antibody diluted 1:1000, washed three times, blocked for 15 min, and incubated for 1 h with goat anti-rabbit alkaline phosphatase-conjugated antibody (Vector) diluted 1:1000. Cells were washed two times, and antibody binding was measured by adding 0.25 ml ABTS (Fisher) to each well and incubating at room temperature for 25 min. Plates were read at 405 nm in a microplate reader (Bio-Rad) using Microplate Manager software.

To evaluate agonist-promoted internalization of WT and mutant CXCR4, HEK293 cells stably expressing Flag-tagged CXCR4 in 24-well plates were washed with serum-free DMEM and then stimulated with 50 nM CXCL12 for 0 to 60 min. The medium was aspirated, cells were fixed, and cell surface expression was analyzed using a rabbit Flag antibody and goat anti-rabbit alkaline phosphatase-conjugated antibody as described above.

### Analysis of CXCR4 degradation

To measure CXCR4 degradation, we followed a previously described protocol (23). Briefly, HEK293 cells expressing stably transfected WT or mutant CXCR4 were initially incubated in DMEM media with 10% FBS containing 10 μg/ml cycloheximide for 10 min at 37 °C. Cells were then incubated in the same media at 37 °C with or without 50 nM CXCL12 for 0, 1, 3, or 6 h. Cells were lysed by addition of lysis buffer on ice and rocked at 4 °C for 30 min, debris was cleared by centrifugation at 14,000 rpm for 20 min, and proteins were resolved by SDS-PAGE and transblotted onto nitrocellulose. Blots were incubated with an anti-Flag polyclonal antibody and analyzed by chemiluminescence. Blots were then stripped and probed using an anti-α-tubulin primary antibody (Sigma). Films were quantified using a LI-COR scanner and normalized for total protein using the α-tubulin blot.

### Statistics

Western blots were quantified on a LI-COR C-DiGit scanner. Data are shown as the mean ± SD with *p* values determined by comparing the data from experimental *versus* control from at least three independent experiments using a one-tailed *t* test in Excel. Where noted, n equals the number of independent experiments that were performed.

## Data availability

All data are contained within the manuscript. Additional information and reagent requests should be directed to the corresponding author.

## Supporting information

This article contains supporting information.

## Conflict of interest

The authors declare no conflicts of interest with the contents of this article.
