# Supplementary material for: Characterization of a new WHIM syndrome mutant reveals mechanistic differences in regulation of the chemokine receptor CXCR4
Source: J Biol Chem. 2021 Dec 30;298(2):101551. doi: 10.1016/j.jbc.2021.101551 (PMC8802859; doi:10.1016/j.jbc.2021.101551)
Supplement: Supplemental Figure S1 [file mmc1.pdf]

Supplementary Figures

Figure S1. **C-terminal amino acid sequences of wild type CXCR4 and known WHIM mutations.** Serine residues in WT CXCR4 that have been identified as phosphorylation sites are in red while mutated residues or altered residues due to a frame shift are underlined. The WHIM mutant, S339fs5, reported in this study as well as the most common WHIM mutant, R334X, are marked with an asterisk.

Figure S1

	310 320 330 340 350
WT CXCR4	**--KFKTSAQHALTSVSRG*SS*LKIL*S*KGKRGGHS*S*VSTESE*SS*SFHSS**
E343K	**--KFKTSAQHALTSVSRGSSLKILSKGKRGGHSSVSTKSESSSFHSS**
E343X	**--KFKTSAQHALTSVSRGSSLKILSKGKRGGHSSVST**
S341fs	**--KFKTSAQHALTSVSRGSSLKILSKGKRGGHSSVPLSLSLQVFTPANTDVKDFFLYDK**
S339fs3	**--KFKTSAQHALTSVSRGSSLKILSKGKRGGHSCFH**
S339fs5*	**--KFKTSAQHALTSVSRGSSLKILSKGKRGGHSFICFH**
S338X	**--KFKTSAQHALTSVSRGSSLKILSKGKRGGH**
G336X	**--KFKTSAQHALTSVSRGSSLKILSKGKRG**
R334X*	**--KFKTSAQHALTSVSRGSSLKILSKGK**
L329fs	**--KFKTSAQHALTSVSRGSSLKIQRKARWTFICFH**
S324fs	**--KFKTSAQHALTSVSRGVQPQDPLQRKARWTFICFH**
